# Pyoderma gangrenosum-like ulceration as a presenting feature of pediatric granulomatosis with polyangiitis

**DOI:** 10.1186/s12969-021-00564-8

**Published:** 2021-06-05

**Authors:** Rotem Semo Oz, Oluwakemi Onajin, Liora Harel, Rotem Tal, Tomas Dallos, Adena Rosenblatt, Lukas Plank, Linda Wagner-Weiner

**Affiliations:** 1 Section of Pediatric Rheumatology, University of Chicago Medical Center, Chicago, IL USA; 2 Section of Dermatology, Department of Medicine, University of Chicago Medical Center, Chicago, IL USA; 3 Rheumatology Unit, Schneider Children’s Medical Center of Israel, Petach Tikva, Israel; Sackler School of Medicine, Tel Aviv University, Petach-Tikva, Israel; 4 Department of Pediatrics, Comenius University Medical Faculty in Bratislava and National Institute of Children’s Diseases, Bratislava, Slovakia; 5 Section of Dermatology and Department of Pediatric, University of Chicago, Chicago, USA; 6 Department of Pathology, Comenius University Jessenius Medical Faculty and University Hospital, Martin, Slovakia

## Abstract

**Background:**

Granulomatosis with polyangiitis (GPA) is an anti-neutrophilic cytoplasmic antibody-associated vasculitis affecting small to medium-sized vessels and involves most commonly the kidneys and the respiratory tract. Skin involvement can be seen in up to 50% of children with GPA and is the initial presenting symptom in 7.7%. Pyoderma gangrenosum (PG)-like ulcers are rarely described as a skin manifestation in GPA and very few cases have been reported previously in children.

**Case presentation:**

We describe 3 new pediatric cases of GPA with PG-like ulcerations. The median age at first symptom was 15 years. Two patients had PG-like ulceration as their initial presentation; additional symptoms eventually led to the diagnosis of GPA 2–24 months later. In 1 case, proteinase 3 (PR3) was negative when first tested, but converted to positive when systemic symptoms emerged; in the other 2 cases PR3 was positive at presentation. All 3 patients had prominent facial lesions. None of the patients responded to treatment with antibiotics or medications commonly used to manage PG, including corticosteroids and cyclosporine. All patients had excellent responses to rituximab. An electronic database literature review was performed and 4 previously reported cases were identified. We assessed the clinical characteristics, serology, and response to treatment of the previously reported and our newly diagnosed cases.

**Conclusion:**

PG-like ulceration is a rare presentation of pediatric GPA which may precede classic systemic GPA symptoms. The predominance of facial ulcer, granulomatous and neutrophilic inflammation on skin biopsy and lack of response to PG treatments are characteristic of GPA-associated PG-like ulcers. Our review suggests that treatment with rituximab may be needed to improve the skin lesions. Recognizing that PG-like ulcerations can occur in pediatric GPA may result in timely diagnosis, appropriate treatment and improved prognosis.

## Background

Granulomatosis with polyangiitis (GPA), formerly known as Wegener’s granulomatosis, is a form of anti-neutrophil cytoplasmic antibodies (ANCA) associated vasculitis [1]. GPA affects small to medium-sized vessels and involves most commonly the kidneys and respiratory tract [2]. Skin involvement has been reported in 36–50% of children with GPA and is the initial presenting symptom in 7.7%. Palpable purpura and acneiform papules are most commonly seen [3, 4]. Rarely, patients with GPA develop pyoderma gangrenosum (PG)-like ulcerations. Very few cases of this dermatologic manifestation have been reported among GPA patients and, more specifically, in pediatric GPA patients. Early recognition of this rare entity and appropriate diagnostic work-up for GPA are essential in order to reduce morbidity and mortality by facilitating disease-specific treatment.

We describe three new pediatric cases initially diagnosed with PG who were later diagnosed with childhood-onset GPA. We also review four previously reported cases in the literature.

## Case 1

**Fig. 1:**
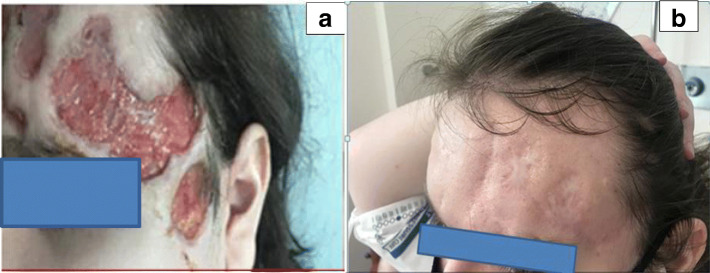
Pre (**a**) and post (**b**) treatment forehead lesions images of a 16-year-old male (patient 1)

**Fig. 2:**
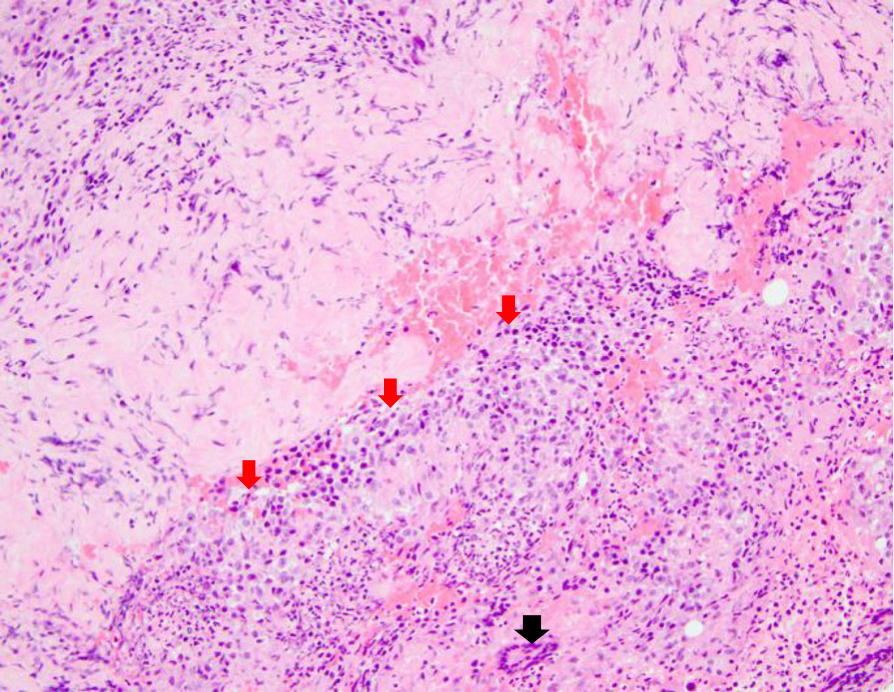
Case 1 Skin biopsy. High power microscopic examination demonstrating palisaded neutrophilic and granulomatous inflammation (red arrows) with multinucleated giant cells (black arrow) and erythrocyte extravasation

A 16-year-old, previously healthy male presented to his primary care physician with acne-like lesions on his face, chest, upper back and arms. He was treated with topical antibiotic and retinoids in addition to oral antibiotics for 2 months; however, the lesions progressed and developed into ulcerations (Fig. 1a). Skin biopsies were performed and initially interpreted as perifollicular inflammation with giant cell reaction and abscess formation consistent with acne. Treatment with oral prednisone, 80 mg daily for suspected PG was initiated with some improvement. A few weeks later, he developed systemic symptoms of cough and sinusitis. Chest CT scan showed central upper lobe nodular consolidating opacity with peripheral ground glass in a peri-bronchial vascular distribution and mild stenosis of some sub-segmental airways. The rheumatology team was consulted and the diagnosis of GPA was made according to EULAR/PRINTO/PRES criteria for childhood GPA based on sinus, pulmonary, and renal (focally crescentic, pauci-immune glomerulonephritis) involvement in addition to elevated inflammatory markers and a positive PR3. Upon further review, the initial skin biopsy was interpreted as palisaded neutrophilic and granulomatous inflammation with multinucleated giant cells and erythrocyte extravasation (Fig. 2). He was treated with plasmapheresis, followed by 2 doses of IV cyclophosphamide and a course of rituximab (two doses of 1000 mg given 2 weeks apart). He was given intravenous (IV) methylprednisolone (1 g/day for 3 days) initially, and then oral prednisone (60 mg/day (0.67 mg/kg/day) tapered off over 6 months). This treatment course, which was chosen based on the extensive pulmonary findings, renal involvement and worsening PG-like ulcerations resulted in significant improvement of the skin ulcerations (Fig. 1b) and his systemic disease. He continues in remission on rituximab maintenance therapy every 6 months.

## Case 2

**Fig. 3:**
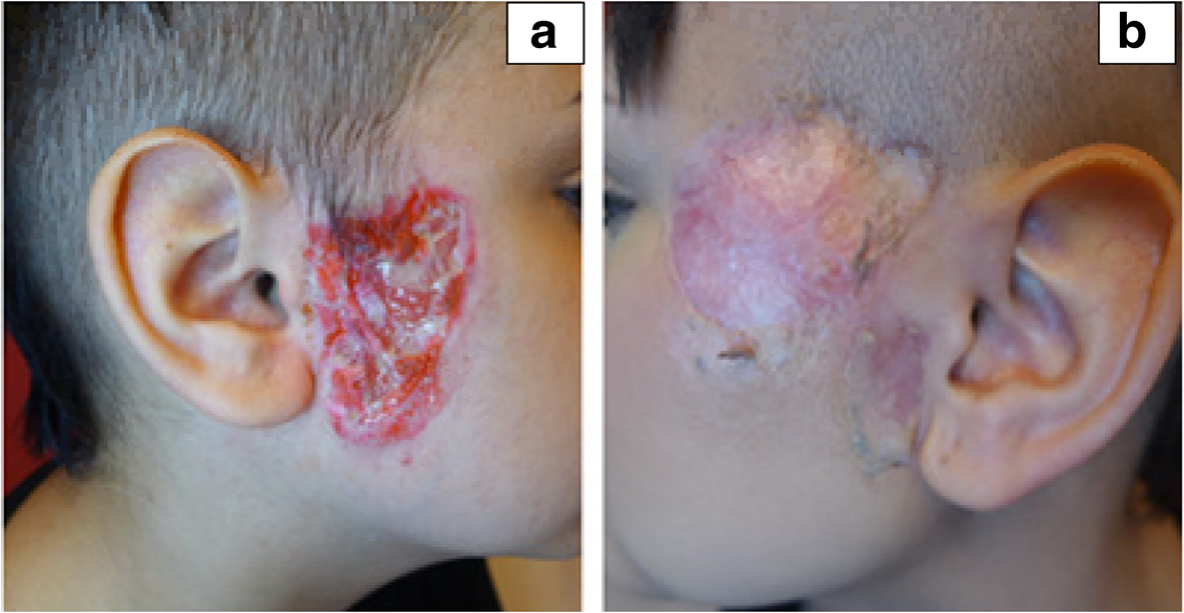
Pre (**a**) and post treatment images of (**b**) facial lesions of a 15 year old female (patient 2) showing poor cosmetic outcome

**Fig. 4:**
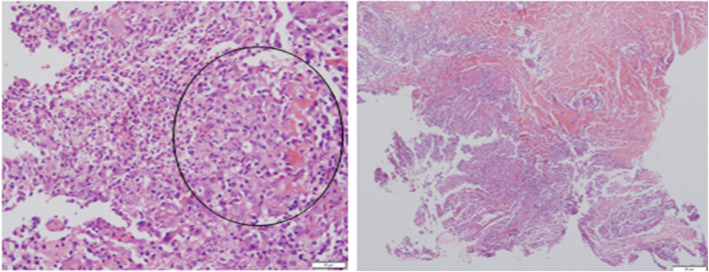
patient 2 skin biopsy. High and low power microscopic examination demonstrating granulomatous and neutrophilic inflammation (black circle) within the deep dermis

A 15-year old female presented with left arm and facial ulcerations (Fig. 3a). An extensive infectious and immunologic work-up was negative, including ANCA serology. Chest x-ray and colonoscopy were normal. She received more than 1 year of various topical and systemic treatments including antibiotics, mycophenolate mofetil, prednisone (maximum dose of 1 mg/kg/day), colchicine and cyclosporine for presumed PG without significant improvement in the lesions. One year after the initial presentation, she developed chronic sinusitis resistant to antibiotic, revealed elevated inflammatory markers and a positive PR3 for the first time. Sinus biopsy showed necrotizing granulomatous inflammation. Skin biopsy was interpreted as granulomatous and neutrophilic inflammation within the deep dermis (Fig. 4). She was treated with pulse methylprednisolone and a course of rituximab, followed by maintenance therapy with rituximab every 6 months. Her treating physician elected to continue rituximab every 6 months for maintenance therapy based on the excellent response to this medication during induction. Since commencement of this treatment regimen, no new skin lesions have appeared. The facial ulcers healed with poor cosmetic result (Fig. 3b).

## Case 3

**Fig. 5:**
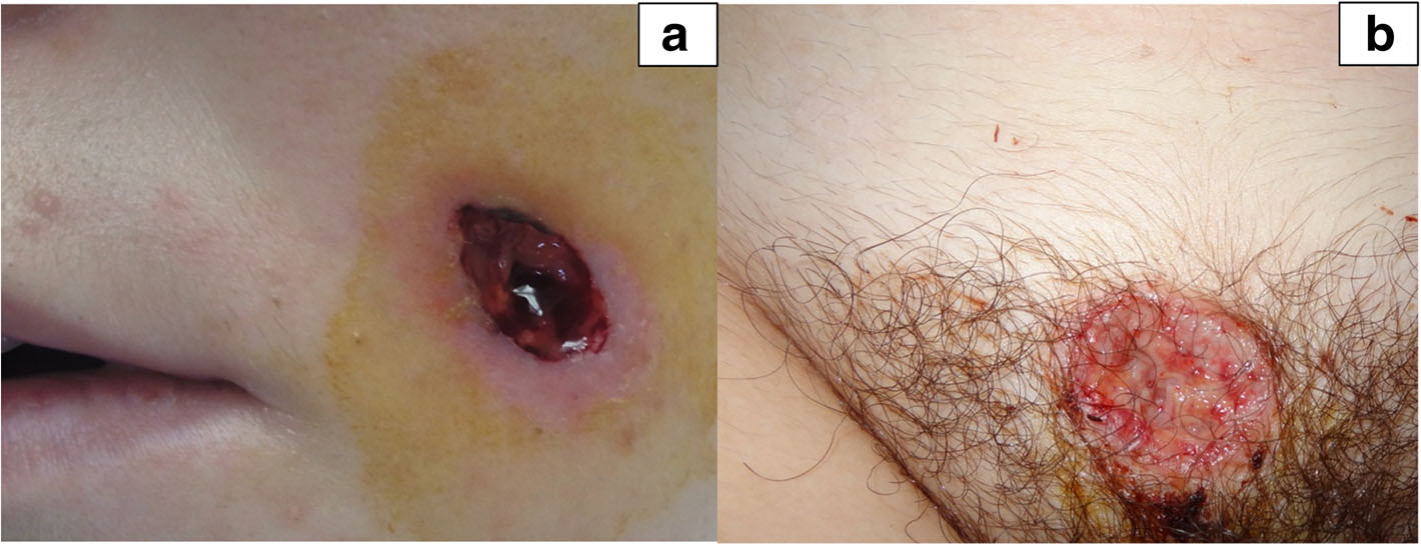
**a-b**: Facial (**a**) and pubic (**b**) ulcerative lesions of a 14-year old female (patient 3)

**Fig. 6:**
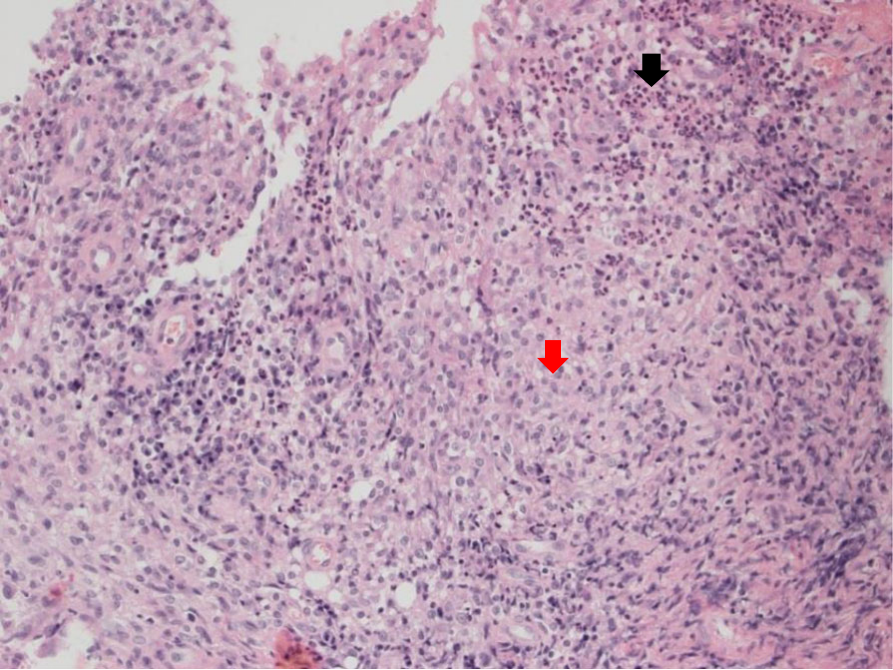
Skin biopsy (patient 3). High power microscopic examination demonstrating granulomatous inflammation comprised predominantly of histiocytes (red arrow), neutrophils (black arrow), few plasma-cells and multinucleated giant cells

A 14-year old female presented with a solitary, painful, nodule located above the left scapula. Three weeks later, she developed a dry cough, hemoptysis and chest pain. Inflammatory markers were increased (ESR 130 mm/hour, CRP 195 mg/L). A homogenous infiltration predominantly of the left lung parenchyma, granulomas, and stenosis of the left main bronchus were found on chest CT. Her poor clinical condition did not allow for a lung biopsy. Empiric antibiotic treatment for suspected pneumonia was ineffective. She developed additional skin lesions on her left forehead, left cheek, posterior aspect of the neck, back and in the pubic area that all progressed into extremely painful, large (up to 7 cm), deep, sharp-edged ulcers with an erythematous and fibrinous base (Fig. 5a, b). A skin biopsy, reviewed by our institution, displayed granulomatous inflammation comprised predominantly of histiocytes, neutrophils, few plasma-cells and multinucleated giant cells (Fig. 6). Given these pathology findings, in addition to lung and sinus involvement and a positive PR3, the diagnosis of GPA was given. Treatment with pulse intravenous methylprednisolone, oral prednisone and monthly IV cyclophosphamide (cumulative dose 5.5 g) did not induce remission of her cutaneous or systemic features. Treatment with rituximab dramatically improved her PG-like lesions and clinical condition; however, she developed a 50% subglottic stenosis and required local treatment with corticosteroid injections and dilation. The currently 20-year old patient’s GPA is in remission on azathioprine and IVIG replacement for hypogammaglobulinemia resulting from persistent B-cell depletion.

## Results of literature review

A search through two electronic databases (PubMed and Medline), using the following keywords: GPA, PG, PG-like lesions/ulceration, Wegener’s granulomatosis, pediatric, identified 4 pediatric cases of GPA with PG-like lesion [5–9]. Clinical characteristics of these cases in addition to our three new cases reported above are presented in Table 1. The median age at first symptom was 15 years with M:F ratio of 5:2. All patients had a positive PR3 (except patient 5 for which ANCA status was not available). In 2 cases, PR3 was negative when first tested, but converted to positive on repeat evaluation when systemic symptoms emerged. Five children had PG-like ulceration as their initial presentation (patients 1, 2, 5, 6, 7), Additional symptoms eventually led to GPA diagnosis 2-24 months later. The ulcerative lesions initially manifested as purpura, nodule or acne in 4 patients. All patients had mainly facial lesions. Additional locations were chest and back. Four of seven patients had renal involvement and 6/7 had upper respiratory tract involvement.

**Table 1:**
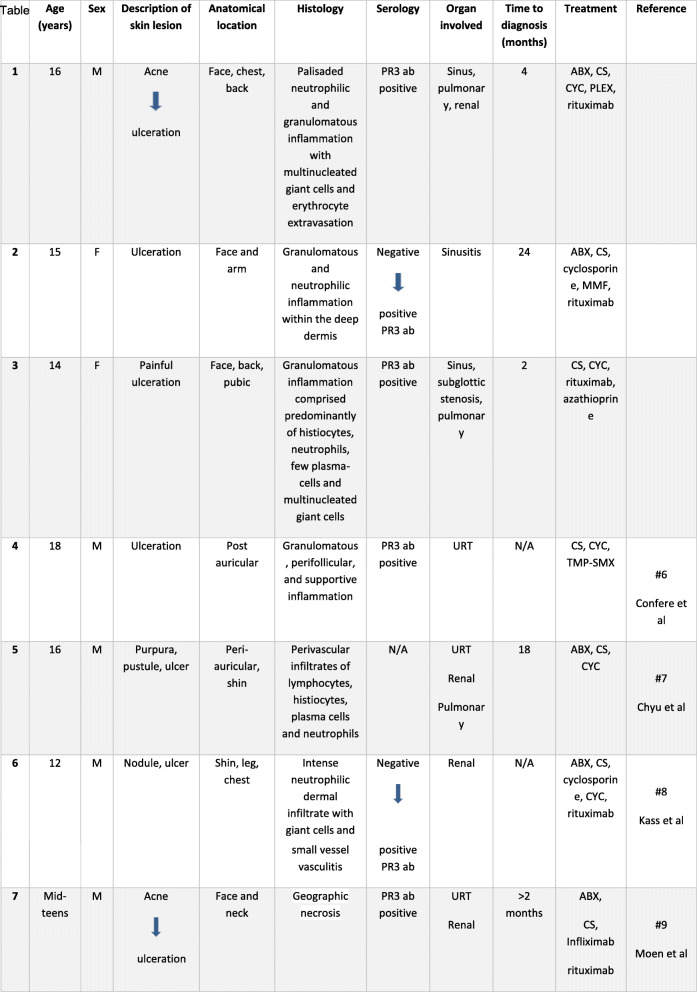
Demographic and clinical characteristics of patients with GPA and PG-like lesions

Abbreviation: *M* male, *F* female, *PR3 ab* proteinase 3 antibody, *N/A* not available, *ABX* antibiotic, *CS* corticosteroids, *CYC* cyclophosphamide, *MMF* mycophenolate mofetil, *PLEX* plasma exchange, *TMP-SMX* trimethoprim/sulfamethoxazole, *URT* upper respiratory tract

## Discussion

Although cutaneous manifestations are common in GPA, PG-like lesions are rarely seen. Frumholtz et al. summarized the cutaneous manifestations of 1553 adult patients with ANCA-associated vasculitis. While 36.7% (273/743) of patients with GPA had cutaneous involvement, PG was observed in only 1.1% of patients [10]. Very few cases of GPA with PG-like lesions have been reported previously, especially in children. We hereby present 3 new cases of pediatric GPA with PG-like lesions, and review these together with the 4 previous pediatric cases found in the literature.

PG is a rare, rapidly progressive, sterile ulcerating neutrophilic dermatosis which usually presents as painful pustules or nodules. It may evolve into necrotic plaques with raised edges and ultimately into deep violaceous ulcers [11–13]. PG lesions most frequently affect the lower extremities and tend to be multifocal and recurrent. Adults aged 20–50 are most commonly affected, while pediatric cases account for approximately only 4% of cases [14]. The diagnosis is based on the typical clinical appearance and accompanying histological findings which can be variable and often non-specific. These include a predominantly neutrophilic inflammatory infiltrate with evidence of necrosis and hemorrhage at the base of the lesion [15, 16]. Approximately 50–70% of PG cases are associated with systemic conditions, particularly autoimmune and auto-inflammatory diseases and malignancies [17, 18]. In the pediatric population, inflammatory bowel disease, juvenile idiopathic arthritis (JIA), Takayasu arteritis and immunodeficiency conditions have been associated with PG [19, 20].

Weenig et al. reviewed medical charts of 240 patients who received the diagnosis of PG and found that 49 patients (20%) had a different final diagnosis including vascular occlusive disease, vasculitis, cancer and infection. The mean lag time to the final diagnosis was 10 months [21]. Ulcerative skin involvement mimicking PG is often termed “pyoderma gangrenosum-like ulceration**”.** There may be a lack of widely used diagnostic criteria, but PG-like ulceration and PG are two separate entities with different histopathologic findings [22, 23]. The histopathologic features of PG-like ulcers in GPA include palisaded neutrophilic and granulomatous dermatitis, necrotizing vasculitis and basophilic collagen degeneration. These histopathologic features are not typically seen in classic PG. The term “PG-like ulceration” is preferred in the setting of systemic findings that are consistent with GPA. The clinical differential diagnosis for PG-like lesions of GPA presenting on the face also includes infections, malignancy (including squamous cell carcinoma, basal cell carcinoma and lymphomas), trigeminal trophic syndrome and factitial ulcer.

The clinical characteristics of the 7 pediatric patients summarized in Table 1 were slightly different from known demography of pediatric GPA patients. The median age at diagnosis was 15 years, which is older than the median pediatric age at GPA diagnosis (12.5–14.5 years) found in previous studies. There was a male predominance 5:2, while previous studies show a female predominance in pediatric GPA patients [24, 25].

Notably, all 7 patients had facial involvement. In contrast_,_ the most common anatomical location of classic PG lesions is the lower extremities, found in 50% of adults. Facial involvement is very uncommon in classic PG [26]. Systematic review of 170 pediatric cases with PG found 46% of patients had disseminated skin disease, 30% had disease limited to the lower extremities and only 3.9% had facial involvement [11]. The atypical anatomical distribution with predilection for facial lesions should increase suspicion of an underlying disease such as GPA.

The common treatment for PG includes topical therapy in milder disease (corticosteroids, dapsone, tacrolimus etc.) and systemic treatment with oral corticosteroids as first line and cyclosporine as a second line treatment for more resistant disease [18, 27]. Our three cases presented above had only minimal improvement on antibiotic and multiple immunosuppressive medications. However, treatment with rituximab led to resolution of active skin disease in all cases. A similar response to rituximab was reported in adults with GPA and PG-like ulceration [22, 23, 28]. Significant scaring remained as a result of the depth of the ulcerating lesions. The initial treatment given to the patients described above not only had a minimal effect on the PG-like lesions, but also may have masked and delayed the systemic presentation of GPA, leading to delayed referral for rheumatology evaluation. Furthermore, it is important to be cognizant that a negative ANCA does not exclude the diagnosis of GPA and additional studies, such as lung and sinus imaging, full renal assessment and possible tissue biopsies, should be considered when GPA is suspected in patients with ulcerative skin lesions. Delay in diagnosis and initiation of appropriate treatment can lead to more extensive skin disease and scarring, as well as increased morbidity and mortality related to the underlying GPA diagnosis.

The limitations of this paper include the small number of patients. We tried to overcome this issue by performing an international multicenter collaboration, as well as the literature review to seek out other pediatric GPA patients with PG-like ulcerations. Additionally, the initial pathology specimens were assessed by different pathologists. However, a dermato-pathologist from one institution reviewed the histopathology images of all 3 patients.

## Conclusion

PG-like ulceration is a unique and rare presentation of pediatric GPA which sometimes can precede the classic systemic GPA symptoms. The predominance of facial ulceration, granulomatous and neutrophilic inflammation on skin biopsy and lack of response to PG treatment are much more common in GPA-associated skin ulceration than in PG. Treatment with rituximab resulted in improvement of both the PG-like ulcerations and other GPA disease manifestations in most of the patients in this review. Recognizing that PG-like lesions can occur in pediatric GPA may result in a timely diagnosis and appropriate treatment, and thus can help improve prognosis.

## Data Availability

The datasets used and/or analyzed during the current study are available from the corresponding author on reasonable request.

## Abbreviations

ANCA: Anti-neutrophil cytoplasmic antibodies
CT: *Computerized tomography*
GPA: Granulomatosis with polyangiitis
IV: Intravenous
JIA: Juvenile idiopathic arthritis
PG: Pyoderma gangrenosum
PR3: Proteinase 3
